# Aspects of Occlusal Recordings Performed with the T-Scan System and with the Medit Intraoral Scanner

**DOI:** 10.3390/diagnostics14131457

**Published:** 2024-07-08

**Authors:** Angelica Diana Popa, Diana Elena Vlăduțu, Adina Andreea Turcu, Daniel Adrian Târtea, Mihaela Ionescu, Cătălin Păunescu, Răzvan Sabin Stan, Veronica Mercuț

**Affiliations:** 1 Department of Prosthetic Dentistry, University of Medicine and Pharmacy of Craiova, 200349 Craiova, Romania; dr.diana_popa84@yahoo.com (A.D.P.); veronica.mercut@umfcv.ro (V.M.); 2 Department of Oro-Dental Prevention, University of Medicine and Pharmacy of Craiova, 200349 Craiova, Romania; 3 Department of Oral Rehabilitation, University of Medicine and Pharmacy of Craiova, 200349 Craiova, Romania; daniel.adrian.tartea@gmail.com; 4 Department of Medical Informatics and Biostatistics, University of Medicine and Pharmacy of Craiova, 200349 Craiova, Romania; 5 Department of IT, University of Medicine and Pharmacy of Craiova, 200349 Craiova, Romania

**Keywords:** occlusal analysis, T-Scan III system, Medit I600 intraoral scanner

## Abstract

Introduction: Dental occlusion refers to the static and dynamic relationships that are established between the teeth of the two arches and is an important factor in the homeostasis of the dento-maxillary system. The objective of the present study was to compare two digital occlusal analysis systems: the T-Scan III system and the Medit I600 intraoral scanner. Materials and Methods: The study was carried out on 20 students from the Faculty of Dental Medicine Craiova, whose dental occlusion was assessed with the T-Scan III system and with the Medit I600 intraoral scanner. Dental occlusion was assessed in the maximum intercuspation position, the edge-to-edge protrusion position, and the edge-to-edge position in right and left laterotrusion. The images of the 2D occlusal contact areas obtained by both methods were converted to .jpeg format and then transferred to Adobe Photoshop CS6 2021 (Adobe Systems, San Jose, CA, USA) for comparison. The recorded data were statistically processed. Results: Analyzing the data provided by the two digital occlusal analysis systems, it was found that the T-Scan III system provided data related to the amplitude of the occlusal forces, the surface on which they were distributed (the contact surface), the dynamics of the occlusal contacts, and the proportion in which they were distributed at the level of the two hemiarches, and the Medit I600 intraoral scanner performed an evaluation of the occlusal interface of the two arches, highlighting the extent of the contact areas with the degree of overlapping of the occlusal components. Although both methods of occlusal analysis recorded the highest values for the maximum intercuspation position, the results could not be compared. Conclusions: The two digital systems provide different data in occlusal analysis. As the T-Scan III system is considered the gold standard for occlusal analysis, more studies are needed to understand the data provided by the Medit I600 intraoral scanner and their significance.

## 1. Introduction

Dental occlusion represents the static and dynamic relationships between the teeth of the maxillary and mandibular arches [1]. The teeth of the two arches establish static relationships during maximum intercuspation at the end of the masticatory cycle and during swallowing or clenching. The teeth can also come into contact during mandibular movements from food incision, trituration, or teeth grinding from bruxism [1]. Balanced dental occlusion is associated with oral health and is considered an important indicator of the functional state of the masticatory system [2,3,4]. Occlusal stability is defined by multiple and simultaneous occlusal contacts, the absence of tooth pain, the absence of periodontal disease, an acceptable vertical dimension of occlusion, age-appropriate tooth wear, no tooth loss, and the presence of harmony between the anterior guide and the occlusal plane [5]. Dental occlusion is a critical factor in the success of any dental restoration [6]. In the case of oral rehabilitation with prosthetic restorations, occlusal balancing must be carried out preoperatively, during the adaptation of the restoration, after its cementation, and even six weeks after cementation in the oral cavity, in order to ensure its integration and functionality within the dento-maxillary system [7]. 

The assessment of dental occlusion can be performed by qualitative and quantitative methods [8]. This should provide data on the locations of occlusal contacts, their durations, and the amplitude of the occlusal forces [2]. The qualitative evaluation of dental occlusion is carried out by means of an occlusal support (articulation paper, wax, silicone) that registers the positions of the teeth and their antagonists. The disadvantages of these qualitative methods are represented by the lack of reproducibility of these records and the lack of objectivity. Quantitative records use various indices to assess dental occlusion. The T-Scan III analysis system, the Dental Prescale system, and the Blue Silicone system make it possible to quantitatively measure the occlusion ratio [2,9,10,11]. 

Occlusal analysis methods can also be divided into conventional methods and digital methods. Conventional methods involve the use of articulating paper, silk strips, occlusal sprays, transillumination, early contact indicators, and occlusal sonography, presenting a number of drawbacks [12,13]. Most of these traditional methods only analyze static occlusion, without considering the mandibular dynamics, including movements from maximum intercuspidation, protrusion, and laterotrusion [8]. Digital methods are based on a software application for the processing of data recorded in a 2D or 3D system to highlight the number of occlusal contacts, their surfaces, and the amplitude of the occlusal forces. Among these systems, the T-Scan III occlusal analysis system has been distinguished, being considered the gold standard in occlusal analysis [12].

The T-Scan III system, developed by Tekscan (Boston, MA, USA), uses a computer to analyze static and dynamic occlusion, both qualitatively and quantitatively, with high precision. The T-Scan III system analyzes the order of the occlusal contacts while simultaneously measuring the percentage changes in the force of these contacts from the time that the teeth first come into occlusal contact until the maximum intercuspation. Moreover, as part of digital occlusal analysis, a number of intraoral scanners include occlusal analysis software. Intraoral scanners allow dentists to obtain three-dimensional digital models of the two separate arches and offer the possibility of the quantitative analysis of dental occlusion [12,14,15]. Such intraoral scanners with occlusal analysis software include the CEREC Omnicam (Detsply Sirona, Charlotte, NC, USA) and, more recently, the Medit I600. Occlusion in CEREC Omnicam is analyzed when the restoration is not yet complete. The occlusion can be digitally adjusted by the system during the design phase. There are a limited number of studies in the literature regarding the use of the CEREC Omnicam intraoral scanner for occlusal analysis [12,16]. The Medit Occlusion Analyzer software (Medit link version 3.1.4—Seoul, Republic of Korea) is an application developed by Medit to simplify dental occlusion analysis within CAD–CAM technology.

The objective of this study was to establish the reliability of the occlusal analysis performed with the Medit Occlusion Analyzer system (Medit link version 3.1.4—Seoul, Republic of Korea), by performing a comparison between the data obtained following static and dynamic occlusion with the T-Scan III digital system and those obtained with the Medit I600 digital system. The null hypothesis stated that there would be no association between the results provided by the two systems regarding occlusal analysis.

## 2. Materials and Methods

The present exploratory research was carried out during 2023 within the Dental Prosthetics Clinic of the University of Medicine and Pharmacy of Craiova. The study was approved by the University and Scientific Ethics and Deontology Commission of the University of Medicine and Pharmacy of Craiova No. 234 on 7 December 2022 and was carried out in compliance with the Helsinki Declaration of 2013 [17].

From the Faculty of Dentistry, 20 students, aged between 20 and 28 years and of both genders, were selected on a voluntary basis, for whom occlusal recordings were performed with the T-Scan III system and the Medit I600 intraoral scanner. The inclusion criteria encompassed students from the Faculty of Dentistry, with intact arches, without signs of temporomandibular disorders. The exclusion criteria included the presence of orthodontic appliances, as they could complicate the data acquisition process, and fixed prosthetic restorations, as they could have modified the occlusal relationships.

In this study, the evaluation of the occlusion reports with the two systems was carried out in the early hours of the morning, initially with the T-Scan III system and then with the Medit I600 intraoral scanner. For the occlusal recordings, the study participants were seated in a dental chair with the head in spine extension. The participants initially received explanations regarding the recording process, and then they underwent the recording procedures. To increase the relevance of the study, recordings with both devices were performed in the positions of maximum intercuspation, protrusion, left laterotrusion, and right laterotrusion.

The Tekscan Inc. T-Scan III system (South Boston, MA, USA) and the Medit I600 intraoral scanner (Medit link version 3.1.4—Seoul, Republic of Korea) were used in this study. The digital T-Scan III system analyzes static and dynamic occlusion and records the occlusal ratios as the teeth of the two arches come into contact, providing data on the amplitude of the occlusal forces, the dynamics of the occlusal contacts, and the duration from the first occlusal contact to the end of the examination. The T-Scan III system includes a sensor with a holder, a handle assembly, the system unit, a software application, and a printer. The sensor is the key component. It is 60 μm thick and composed of a polyester film. The sensors are available in two sizes (large and small). The large sensor is used for arches up to 66 mm in width and 56 mm in length and contains 1370 landmarks, and the small sensor is used for arches up to 58 mm in width and 51 mm in length with 1122 landmarks. The “Evolution Handle (EH-2)” collects data from the sensor and sends them to the computer for processing [18]. The recorded data are presented in 2D or 3D or as a dynamic movie. The system can operate in two modes: time analysis and force analysis. Time analysis provides information on the location and sequence of occlusal contacts, showing, in a different color, the locations of the first, second, and third or more contacts. Force analysis provides the operator with data on the location and relative force of tooth contact [19].

The Medit Occlusion Analyzer (Medit link version 3.1.4—Seoul, Republic of Korea) is an application in the Medit I600 intraoral scanner, developed to simplify the examination of occlusal relationships. The system has the following main technical features: a scanning frame rate of up to 70 FPS, imaging technology consisting of 3D video technology in motion, 3D full color streaming capture, adaptive antifogging, and accuracy for the whole arch of 10.9 μm ± 0.98. The Medit Occlusion Analyzer can automatically analyze the occlusal contacts between the maxilla and mandible and display the results through a color map.

### 2.1. Recording Technique

Before recording with the T-Scan III, the sensor handle was connected to the computer and the program started. The correctly sized sensor for the arcade was selected. The recording handle with the sensor and the arch holder was placed between the patient’s maxillary central incisors. To calibrate the T-Scan III system, the first recording was performed while the participant performed a maximum intercuspid closure. The sensor was placed in the oral cavity and the participant was instructed to bite for up to 3 s with maximum force in maximum intercuspation. When this was confirmed on the screen, the participant opened their mouth, and the sensitivity adjustment was obtained by using the Sensitivity Adjustment Selection tool (Adobe Photoshop CS6 2021, Adobe Systems, San Jose, CA, USA) to make the sensor more or less sensitive to bite forces. After this, the recordings of the maximum intercuspation position, protrusion, and left and right laterotrusion were performed. Recordings were initiated by pressing the button on the recording handle. The patient was asked to close their mouth in maximum intercuspation and then perform the right and left horizontal protrusion and laterotrusion movements. For this, the sensor was inserted into the oral cavity so that its support was centrally aligned with the midline of the upper central incisors. After pressing the handle button, the arcade model was automatically created on the screen. It should be noted that this model provides an approximation of the patient’s arch and therefore there are approximations as to the exact location of the contact on the screen. The recording with the Medit I600 intraoral scanner was performed immediately after the T-Scan III recording, without changing the head position.

### 2.2. Scanning Technique

The camera was inserted into the oral cavity with a spacer. Calibration was performed before each scanning session according to the manufacturer’s instructions. To scan the maxillary arch, it started from the left distal side with the occlusal surface. It was continued by a zig-zag movement in the frontal area to the contralateral part of the arch. It then continued with the oral part of the arch and later with the vestibular part. Images of the maxillary arch and mandibular arch were obtained in this way.

For the recordings of the maximum intercuspation position, a zig-zag movement was performed with the scanner positioned on the vestibular side, along the two arches positioned in maximum intercuspation. For both types of recordings for protrusion movements and right and left laterotrusion, the recordings were performed in the form of films, from which print screens were obtained corresponding to the edge-to-edge protrusion positions, edge-to-edge in the right laterotrusion position, and edge-to-edge in the left laterotrusion position. While scanning the maximum intercuspation (MI) position, the participants were instructed to bite with the maximum force.

In both occlusal recording techniques, interdental contact data were defined with a blue to red color scale, where the blue color represented low values and the red color represented high values. Areas of acceptable tolerance were highlighted in green.

### 2.3. Image Processing Chain

The images of the 2D occlusal contact areas obtained by both methods were converted to .jpeg format and then transferred to Adobe Photoshop CS6 2021 (Adobe Systems, San Jose, CA, USA) for comparison. The number of pixels from the contact areas of the two arches for each participant under study was obtained by processing and analyzing the images in the Adobe Photoshop CS6 2021 graphics processing program. Adobe Photoshop is a software program used to edit digital images on a computer and is aimed especially at professionals in the field. Adobe Photoshop, as it is known today, is the spearhead of the digital image, photo, print, video, and web editing software on the market. Photoshop is a program with an intuitive interface that allows a multitude of changes that are currently needed by professionals and includes features related to brightness and contrast edits; color; focus; applying effects to the image or to areas (selections); retouching degraded images; an arbitrary number of color channels; support for 8-, 16-, or 32-bit color channels; third-party effects, etc. Thus, it was possible to analyze the images differently, depending on the color of the contact areas, resulting in the centralizing table containing, for each patient, the number of pixels related to the 3 basic colors: red, green, and blue.

The images obtained from the two systems were previously edited in the sense of removing areas of no interest (crop), resizing them, and adjusting the resolution in order to standardize the differences resulting from their acquisition with different systems. To extract the weight of a certain color, the color selection tool (Color Range, Adobe Photoshop CS6 2021, Adobe Systems, San Jose, CA, USA) was used, with which all three colors were selected in turn. The characteristics of the contact areas in both systems have the same color code, with areas of heavy contact being represented by red, areas of normal contact by green, and areas of fine contact by blue. The selection of the 3 colors was made with a tolerance of 10%.

**Figure 1:**
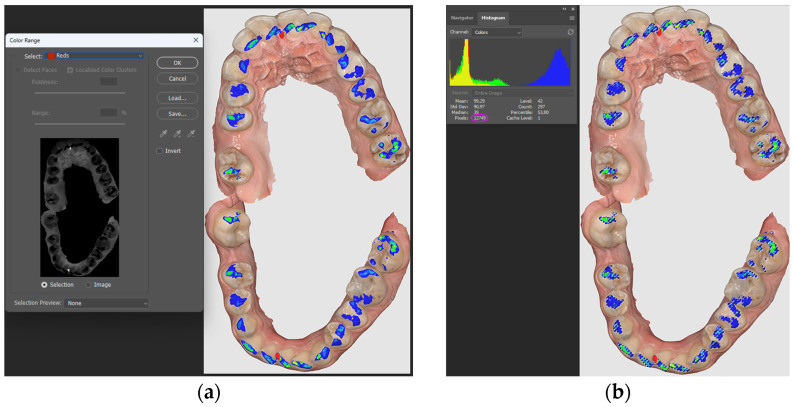
(**a**) Record of occlusal contacts in maximum intercuspation; (**b**) color histogram.

After the selection, it was possible to analyze the histogram related to the image (Figure 1a,b), collecting in the table the number of pixels for each color. Finally, the overlay of the images was also attempted in a 3D CAD processing program, namely AutoCAD 2021, resulting in an overview of the contact areas. AutoCAD is a product of Autodesk and is currently the most popular computer-aided design program. Using the AutoCAD application, users can model objects from the real world (3D) and create technical drawings and graphic representations (2D). It is used as a working tool in the drawing and design activity of engineers, architects, technicians, and engineering students.

The recordings were performed for all participants with the same T-Scan III and Medit I600 devices, and the same software application was used for data processing. All data resulting from the occlusal recordings were entered into an Excel table and processed statistically. The statistical processing of the data was carried out for each device used and subsequently the data obtained with the two devices were compared.

### 2.4. Statistical Analysis

The primary data obtained in this study were centralized using the Microsoft Excel 365 program (Microsoft, San Francisco, CA, USA) and included the values of the measurements performed with the two biological data acquisition instruments, the T-Scan III and Medit I600. The Microsoft Excel application was also used to develop the data presentation graphs included in the Section 3. Detailed descriptive statistical data processing for the entire data set was performed using the IBM Statistical Package for the Social Sciences (SPSS) program, version 20.0 (IBM Corp., New York, NY, USA). The data were initially analyzed to check for normality and homogeneity, by means of Shapiro–Wilk tests for normality of distribution, as well as Levene’s test for the homogeneity of variances. All variables in the study were continuous and were expressed as the mean ± standard deviation (SD) for normally distributed data or the median when the condition of normality was not met. Results correlations were performed using Kendall’s tau-b test, a nonparametric test used to assess the association between two continuous variables. Results were considered statistically significant at α = 5% and *p* < 0.05.

## 3. Results

Evaluating the results obtained through the two recordings, it can easily be seen that the T-Scan III system provided data related to the amplitude of the occlusal forces, the surface on which they were distributed (the contact surface), the dynamics of the occlusal contacts, and the proportion in which they were distributed at the level of the two hemiarches. According to the color code reflecting the amplitude of the occlusal forces, the red color indicates high-amplitude occlusal forces, the green color indicates normal occlusal forces, and the blue color indicates minimal-intensity occlusal forces.

Evaluating the results obtained with the Medit I600 intraoral scanner, it was found that an evaluation of the occlusal interface of the two arches was carried out, highlighting the extent of the contact areas with the degree of overlapping of the occlusal components. The extent of the occlusal contact surfaces was measured in pixels, and the degree of overlapping of the cusps in the pits was expressed in color. Thus, the red color showed an overlap greater than 0.1 mm, the green color showed normal occlusal contact, and the blue color showed light occlusal contact.

Evaluating the values obtained and correlating them with the occlusal morphology, it was found that the highest values with the Medit I600 scanner in maximum intercuspation were obtained for participants with a primary occlusal morphology (no tooth wear). Therefore, we consider that the values reflect the degree of cuspidation of the teeth.

The surfaces of these colored areas were estimated by the number of pixels.

**Figure 2:**
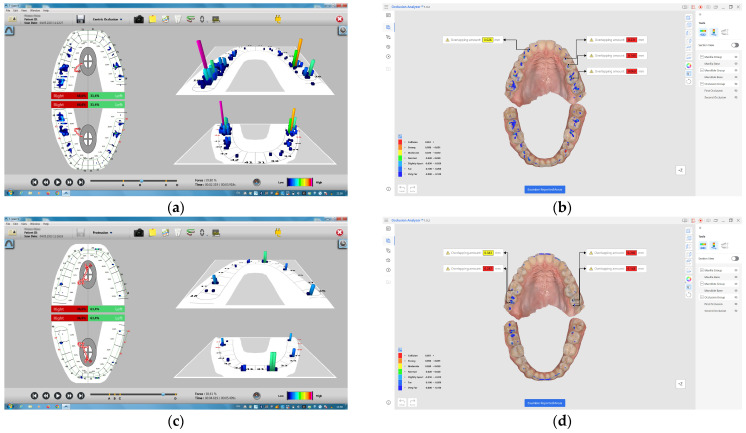

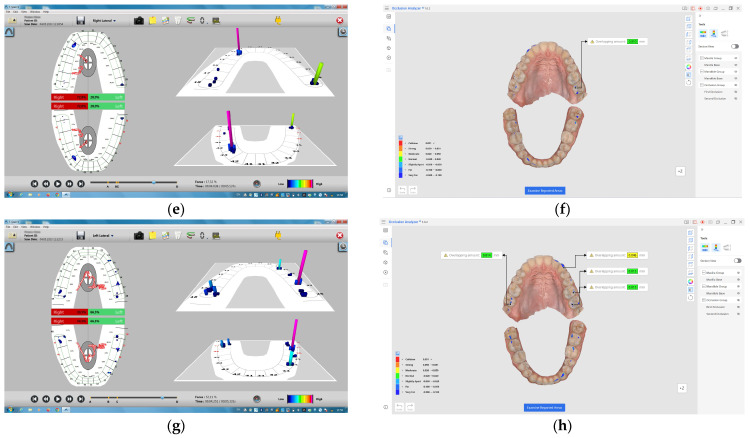
(**a**) Recording of maximum intercuspation position with the T-Scan III system; (**b**) recording of the maximum intercuspation position with the Medit I600 intraoral scanner; (**c**) recording of the edge-to-edge protrusion position with the T-Scan III system; (**d**) recording of the edge-to-edge protrusion position with the Medit I600 intraoral scanner; (**e**) recording of the edge-to-edge right laterotrusion position with the T-Scan III system; (**f**) recording of the edge-to-edge right laterotrusion position with the Medit I600 intraoral scanner; (**g**) recording of the edge-to-edge left laterotrusion position with the T-Scan III system; (**h**) recording of the edge-to-edge left laterotrusion position with the Medit I600 intraoral scanner.

Aspects of the occlusal recordings obtained by the two methods are presented in Figure 2a–g.

Following the quantitative evaluation of each registration with the T-Scan III system, based on the number of pixels, the data obtained for the 20 participants were as presented in Table 1.

### 3.1. Analysis of the Results Obtained with the T-Scan III System

All four variables analyzed through the T-Scan III system were expressed numerically by a value that represented the number of pixels of a certain color (red, green, blue). Table 2 includes the centralization of their main values.

The maximum intercuspation position showed the most points of contact, for all three colors. All 20 participants in this study had both points of maximum intensity (expressed by the color red) and minimum intensity (the color blue) and areas of acceptable tolerance (the color green), adding up to a total of 261,191 pixels. The minimum number of maximum-intensity pixels was nine and the acceptable tolerance was 16.

For the maximum intercuspation position, minimum-intensity contact points predominated, with 97.65% of the total points recorded.

**Figure 3:**
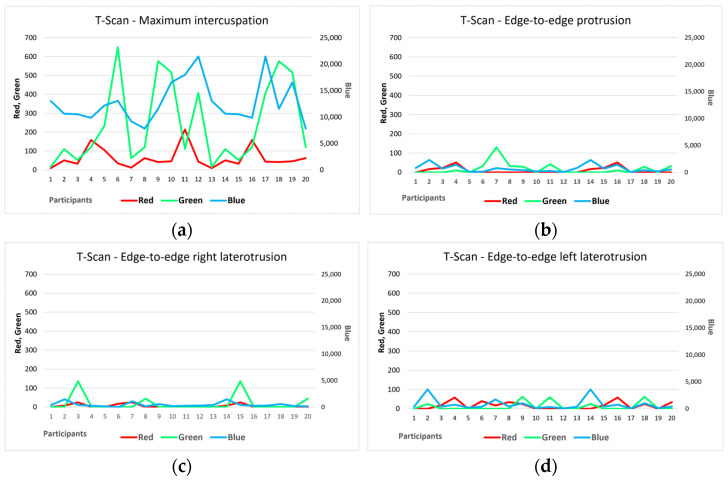
The number of red, blue, and green pixels obtained from the investigation with the T-Scan III system for (**a**) the maximum intercuspation position; (**b**) the edge-to-edge protrusion position; (**c**) the edge-to-edge right laterotrusion position; (**d**) the edge-to-edge left laterotrusion position.

From Figure 3a, a similar trend can be observed for the number of red and blue pixels and, therefore, the areas of maximum and minimum intensity, but without a statistically significant association.

Regarding the analysis of the edge-to-edge protrusion position, only six participants (30%) showed maximum-intensity contact points, and almost half showed acceptable tolerance contact points (nine participants, 45%). Points of minimal intensity were presented by all participants, with seven participants (35%) having only points of this type. At the level of the entire study group, contact areas of minimum intensity prevailed (blue color, 13,900 pixels in total), with a maximum area of 5.45% of the total contact points recorded in the case of the maximum intercuspation position. Figure 3b does not reflect any trend in the number of pixels of different colors.

The analysis of the edge-to-edge right laterotrusion position reflected that more than half of the participants included in the study group showed only contact areas of minimal intensity (12 students, representing 60%), so their investigations did not contain pixels of a red or green color. Specifically, only six participants (30%) showed maximum-intensity contact points, while only four (20%) showed points in the accepted tolerance range. From the entire study group, only two participants (10%) presented all three types of contact points, and only one participant presented only red pixels, i.e., only pixels corresponding to areas of maximum intensity.

Figure 3c shows the evolution of the number of pixels of different colors for all 20 participants, but there are no associations between the variables.

Regarding the analysis of the edge-to-edge left laterotrusion position, 15 participants (75%) presented only contact points of maximum or minimum intensity (or both), without also presenting contact areas with an intensity within the acceptable tolerance range. Moreover, there were only two participants in the entire study group (10%) who had values recorded for all three contact zones of varying intensity, with the rest of the participants showing only one or two contact zones. Contact areas of minimal intensity (blue) were recorded for all 20 participants. Figure 3d shows the evolution of the number of pixels of different colors for all participants, but there are no associations between the surfaces of the contact areas.

### 3.2. Analysis of the Results Obtained with the Medit I600 Intraoral Scanner

The same four variables previously analyzed by means of the T-Scan III system were also analyzed by means of the Medit I600 intraoral scanner, being expressed numerically by a value that represented the number of pixels of a certain intensity (highlighted with different colors: red, green, blue). Table 3 includes the centralization of their main values. It is observed that there are fewer 0 values recorded for the analysis of the contact areas, for the parameters analyzed in the present study.

**Figure 4:**
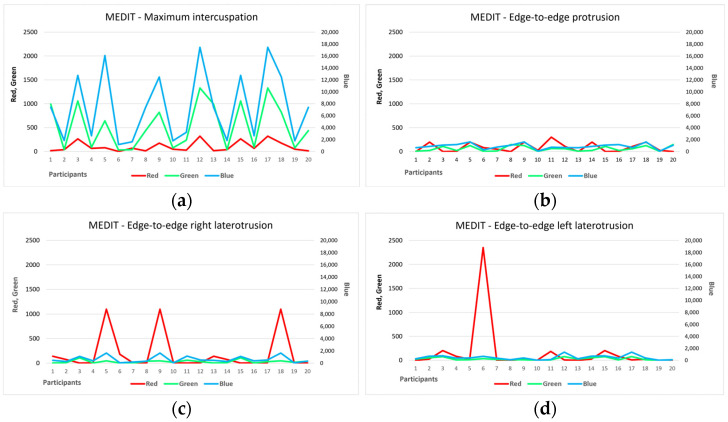
The number of red, blue, and green pixels obtained from the Medit I600 investigation for (**a**) the maximum intercuspation position; (**b**) the edge-to-edge protrusion position; (**c**) the edge-to-edge right laterotrusion position; (**d**) the edge-to-edge left laterotrusion position.

Additionally, in the case of the Medit I600 investigation, the maximum intercuspation position showed the most points of contact for all three colors. Only 19 participants in this study (95%) had both points of maximum intensity (expressed by red color) and minimum intensity (blue color) and intensity zones within the acceptable tolerance limits (green color), adding up to a total of 162,208 pixels. For this position, a single value of 0 was recorded for the areas of maximum intensity, for a participant who also presented the smallest area of intensity contact within the acceptable tolerance range (30 pixels) but also the smallest area of minimum-intensity contact (1165 pixels). From Figure 4a, a similar trend can be observed for the number of green and blue pixels, i.e., the areas of minimum intensity and the limits of acceptable tolerance, but without a statistically significant association.

Regarding the analysis of the edge-to-edge protrusion position, six participants (30%) did not show any maximum-intensity contact points at all. Contact areas with an intensity within the acceptable tolerance range were recorded for all 20 study participants, even if they were sometimes relatively small (three or four pixels), which was also true for the minimum-intensity contact areas. Figure 4b does not reflect similar trends in the number of pixels of different colors.

The analysis of the edge-to-edge right laterotrusion position indicated also that six participants (30%) had no maximum-intensity contact points at all. Among them, for four participants, the value 0 was also recorded in the case of the analysis of the contact areas with the maximum intensity in the edge-to-edge protrusion position. Otherwise, only positive values were recorded, although six participants (30%) showed very small values (1–3 pixels) for contact areas with an intensity within the acceptable tolerance range (green color). Figure 4c does not reflect similar trends in the number of pixels of different colors.

Regarding the analysis of the edge-to-edge left laterotrusion position, seven participants (35%) presented only contact points of minimal intensity or within the acceptable tolerance range (or both), without presenting contact areas of maximum intensity. Moreover, there was only one participant in the entire study group (10%) who only had contact areas with minimum intensity, but even these were relatively small, totaling only 20 pixels (minimum recorded as the number of pixels in this category). The contact areas with an intensity within the acceptable tolerance range had small areas, with a maximum of 78 pixels across the entire study group. Figure 4d shows the evolution of the number of pixels of different colors for all participants, but there are no associations between the surfaces of the contact areas.

**Figure 5:**
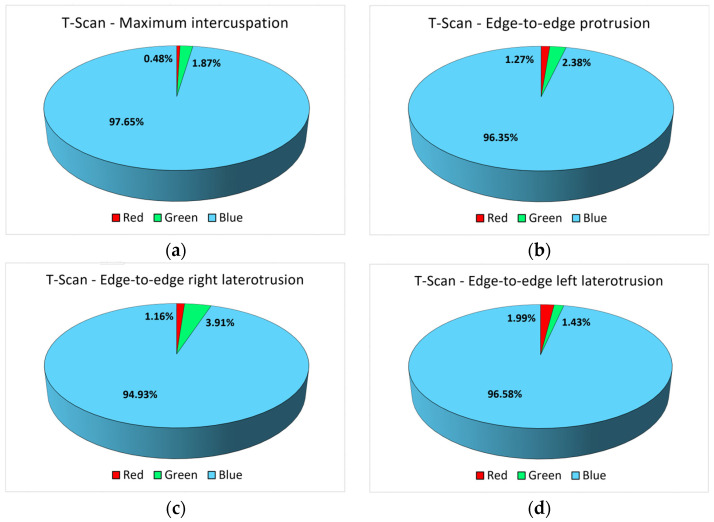
The percentage distribution of red, blue, and green pixels for the 4 types of positions/movements (T-Scan III): (**a**) maximum intercuspation; (**b**) protrusion; (**c**) right laterotrusion; (**d**) left laterotrusion.

**Figure 6:**
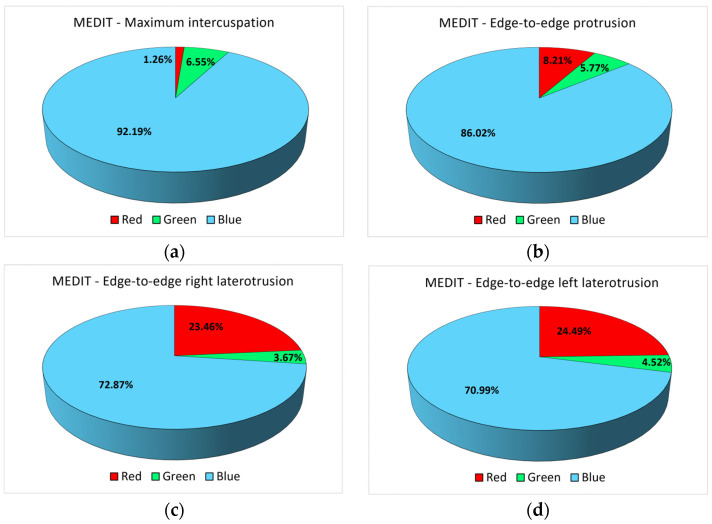
The percentage distribution of red, blue, and green pixels for the 4 types of positions/movements (Medit I600): (**a**) maximum intercuspation; (**b**) protrusion; (**c**) right laterotrusion; (**d**) left laterotrusion.

The comparative analysis between the two methods of occlusal analysis showed that the largest surfaces of the contact areas were recorded for the maximum intercuspation position, with the other positions of the ends of the mandibular movements showing clearly smaller contact surfaces (Figure 3 and Figure 4). The distribution of the surfaces is highlighted in Figure 5 and Figure 6, for each position, motion type, and data acquisition instrument.

For the T-Scan III system, similarity in the distribution of pixels with different intensities is observed for the four types of positions/movements. Blue pixels clearly predominate, reflecting contact areas of minimal intensity.

In the case of the Medit I600 intraoral scanner, there is an increase in the percentage corresponding to the maximum-intensity contact areas (highlighted in red), from the position of maximum intercuspation to the edge-to-edge protrusion position to the types of laterotrusion movement, associated with a decrease in the corresponding percentage of minimum-intensity contact areas (highlighted in blue), also in the same order of variables. The percentage of contact areas with an intensity within the limits of accepted tolerability varies between 3.67% and 5.78%, without showing a clear trend of evolution. Moreover, for the Medit I600, blue pixels clearly predominate, reflecting contact areas of minimal intensity.

The edge-to-edge right laterotrusion position shows the smallest contact areas of minimum intensity for all four variables studied, with a maximum of 1467 pixels for the entire study group, as well as the smallest areas of maximum intensity, with a minimum of 25 pixels for the entire study group.

Regarding the edge-to-edge left laterotrusion position, for this study variable, the lowest maximum number of pixels of tolerable intensity—in the green color (62 pixels)—was recorded.

**Figure 7:**
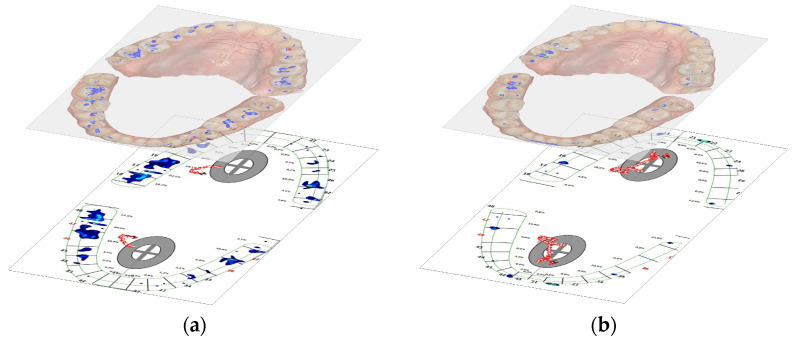

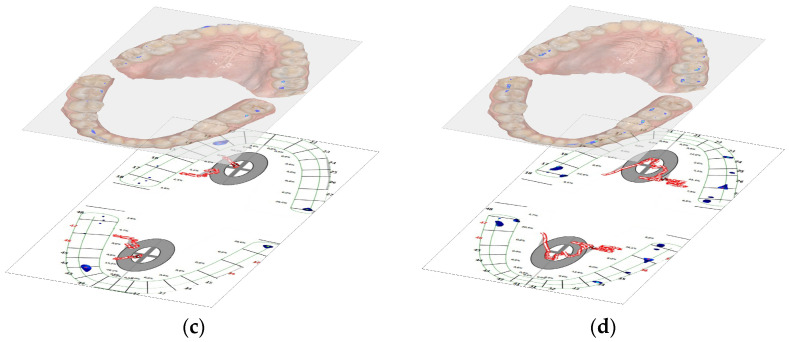
Overlap of images obtained in (**a**) maximum intercuspation position; (**b**) edge-to-edge protrusion position; (**c**) edge-to-edge right laterotrusion position; (**d**) edge-to-edge left laterotrusion position.

The overlap of the images obtained by the two methods of occlusal analysis by means of the CAD 3D processing program shows that the occlusal contact areas identified by the two methods correspond in distribution at the level of the teeth, but, as previous data have shown, their surfaces do not correspond in extent (Figure 7a–d).

## 4. Discussion

Occlusal analysis plays an important role in the evaluation, diagnosis, and application of the occlusal scheme in prosthetic treatment [20,21], orthodontic treatment [22], and other fields of dentistry [2,23]. Regarding the role of occlusion in the optimal functioning of the dento-maxillary system, Okeson stated that occlusal factors, trauma, deep pain, emotional stress, and parafunctions are etiological factors of temporomandibular dysfunction [24]. Besides the specific manifestations of temporomandibular disorder, it is believed that most failures in prosthetic treatment are due to the occlusal scheme. Christensen [6] showed that when he modified the patient’s occlusal scheme through prosthetic treatment, the failure of the prosthetic treatment occurred most of the time. Christensen also stated that following the patient’s occlusal scheme is the key to success in prosthetic treatment, as nature rarely makes mistakes [6].

In this context, a thorough analysis of the occlusion is required before starting the prosthetic treatment to highlight the occlusal disharmonies that must be eliminated during the pre-prosthetic treatment, to record the occlusion in order to reproduce it in the laboratory for the realization of the prosthetic restoration, and, after this, to ensure its integration and functionality within the dento-maxillary system [25].

Among all methods of dental occlusion assessment, the T-Scan III stands out in terms of accuracy and is currently considered in several studies to be the gold standard in dental occlusion assessment [26]. The T-Scan III system for computerized occlusal analysis was developed by Maness in 1987 and provides real-time measurements of the occlusal forces via an intraoral sensor [27]. The first generation of sensors (G1), developed in 1987, has benefited from many changes in design and improvements in the recording capabilities based on clinical studies. The latest generation of sensors is very fine, more sensitive, and thinner (105 μm) than previous sensors [28]. The original design of the T-Scan III system has been modified and improved in both its software and hardware to achieve the current version of the T-Scan III system. The software uses a graphical interface. The program processes the recorded data and displays the acquired values in 2D or 3D color graphics. In the 2D graphics, the occlusal contacts are visualized as colored outlines on the dental arches. In the 3D graphics, the recorded occlusal contacts are visualized as columns with different colors and heights, depending on the amplitude of the occlusal forces. Studies regarding T-Scan III applications in the analysis of occlusal relationships have been conducted by Kerstein RB et al., who found the T-Scan III system to be a highly accurate technique for the study and analysis of occlusal relationships [29]. Koos claimed that the T-Scan III has some advantages in terms of accuracy, reproducibility and the visualization of dental arches [30]. Bozhkova, in 2016, stated that the T-Scan III system enables the accurate determination and correct assessment of the time sequence and magnitude of the occlusal forces by converting qualitative data into quantitative parameters and allowing their digital display [31].

T-Scan III evaluation is a useful clinical method that allows the unbiased assessment of occlusal relationships by an operator. It has applicability in dental prosthetics, in periodontics, in orthodontics, after orthognathic surgery, and in the treatment of bruxism [18,32,33,34,35]. However, due to its high costs, T-Scan III assessment is used more in research and less in current dental practice, perhaps also because the evaluation of dental occlusion is not considered to have major importance in the dental therapeutic practice. On the other hand, other digital technologies are becoming more frequently used in dental practice, especially in recent years, after the onset of the COVID-19 pandemic. It is specifically focused on CAD–CAM technologies with optical impression devices (intraoral scanners).

Intraoral scanners have a wider range of indications and applications in dentistry and their performance has been improved [36,37]. Thus, they can be used in the production of conventional prostheses or with implant support, the guided placement of implants, orthodontic diagnosis, the planning of orthodontic treatment, and the planning of orthognathic surgery [38]. Newer generations of intraoral scanners have higher scanning speeds and better accuracy [39]. Moreover, the scanner handpiece has become smaller, which makes it easier to handle. Intraoral scanners offer a digital alternative to diagnostic and final cast models and to the occlusal positions through virtual occlusal records [40]. Most intraoral scanners provide results comparable to those of high-precision impression materials such as polyethers and polyvinyl siloxanes when considering a segment of dental arches [41]. Complete scans of the dental arches can induce larger errors, which raises concern in the case of implant-supported prostheses and in the case of orthodontic treatment [42].

The first intraoral scanner (IOS) was created in the 1980s [43]. This technology continued to be developed, and, in 1987, the first intraoral scanner (IOS) was introduced in the dental market [44,45]. Sirona Dental Systems LLC (Charlotte, NC, USA) introduced this scanner into the CEREC^®^ to add value in restorative dentistry. After this, many other manufacturers introduced multipurpose IOSs to the market, aiming at several branches of dentistry, including orthodontic purposes [46]. IOSs adopt non-optical technologies in which data are captured through the scanning handpiece and then transmitted to the workstation and displayed on a monitor. These technologies include confocal imaging, triangulation, and 3D motion video [47]. Previously, the conventional impression had to be cast in the laboratory to obtain a physical model, and then an optical scanner was used to obtain the digital model. The digitalized model was subsequently processed using CAD–CAM systems [48]. Since then, IOSs have benefited from significant technological development in terms of software and hardware, becoming very common in dental practice [49]. Zarbakhsh, in 2021, stated that these improvements have allowed IOSs to be as accurate as conventional impressions [50].

Regarding the present study, the results showed significant differences for both methods of evaluating dental occlusion, with the only similarity between the two methods being that the highest values were recorded in the maximum intercuspation position. In fact, the maximum intercuspation position is considered the force position of the jaw, in which the maximum dental contacts are formed between the two arches.

The attempt to overlap the images obtained by the two methods gave relative results since the size of the obtained images varies depending on the used system [12]. In this study, the 3D image obtained with the Medit I600 intraoral scanner was manually overlapped onto the 2D T-Scan III image; therefore, no objective assessment could be performed. The obvious differences obtained between the measurements of the two digital systems can be attributed to the different evaluation parameters and the different pixel sizes between them [12].

In 2020, Sutter B [51], referring to a study conducted by Dias in 2020 [52], where he attempted to obtain correlations between occlusal markings created with articulating paper, recordings of occlusion by optical scanning, and recordings of dental occlusion with the T-Scan III system, showed only a weak correlation, claiming that such a comparison is similar to comparing “apples to oranges”. In other words, although the three techniques in Dias’ study had the same objective, the parameters provided were different. The same situation was noted in the present study.

While the T-Scan III system provided data on the locations of the occlusal contacts and their dynamics, the amplitude of the forces, and their distribution at the level of the dental arches, the Medit I600 intraoral scanner provided data on the locations of the occlusal contacts and the degree of overlapping of the occlusal elements. It can be understood, based on the results obtained by optical scanning and following the occlusal morphology of the analyzed dental arches, that the results obtained by optical scanning are more closely related to the degree of cuspidation of the teeth (the occlusal morphology, reflecting the degree of overlapping) and less to the occlusal contacts.

In fact, the accuracy of intraoral scans is dependent on a number of factors. Revilla-León M et al., in 2023, stated that the factors that can reduce the accuracy of intraoral scanning are [53] calibration [54], the technology used [55,56], the ambient lighting conditions [57], the scanning pattern [58], the extension of intraoral digital scanning [59], the operator’s experience [60], the characteristics of the scanned surface [61], and the humidity conditions [62]. Regarding the accuracy of occlusion registration with intraoral scanners, several factors can affect such an assessment, such as the optical properties of the materials scanned in the oral environment. The optical properties of the scanned materials determine how the light is reflected and refracted. Higher translucency or more reflective materials such as ceramics, zirconia, resins, and metals may result in lower scanning accuracy [61,63]. In addition to these factors that influence the accuracy of intraoral scans in general and the intermaxillary relationships in particular, the performance of various types of intraoral scanners was also studied.

The study by Revilla-León M et al., in 2023 [53,54], also evaluated the differences in the recording of the centric relation by a conventional method, four intraoral scanners, and the jaw tracking Modjaw system. The results of the study showed the best results in terms of correctness for the iTero (iTero Element 5D; Align Technology, Tempe, AZ, USA), Modjaw (Modjaw, Villeurbanne, France), and TRIOS4 (3SHAPE, Copenhagen, Denmark) systems. The i700 device (i700, wireless; Medit, Seoul, Republic of Korea) had the lowest accuracy and precision values, followed by the Primescan system (Dentsply Sirona, Charlotte, NC, USA). Another study by Bostancıoğlu in 2021 compared the performance of the T-Scan III system with that of the CEREC Omnicam system (Sirona Dental System, Charlotte, NC, USA) [12]. The results of the study demonstrated that the CEREC Omnicam system can also perform clinically acceptable occlusal analysis but the data were only sufficient to evaluate strong occlusal contact, as compared to the T-Scan III, which could also evaluate light occlusal contact [12]. Moreover, the TRIOS system (3 Shape, Copenhagen, Denmark was compared with the T-Scan III Novus system (Tekscan, Norwood, MA, USA 2018) [64] in terms of occlusal analysis. The results obtained led the authors to state that intraoral scanning is not a reliable method for the recording of occlusion and that the information obtained is not incorrect but provides insufficient data.

It can be appreciated, on the basis of the study carried out within this research and on the basis of data from the specialized literature, that the results obtained with the Medit I600 system reflect the lower performance of intraoral scanners in terms of occlusal analysis. In practice, the Exocad software is used for the occlusal modeling of prosthetic restorations, which presents a module dedicated to occlusal relationships (the Auto Articulator Module). However, even this software application presents certain deficiencies regarding the functional occlusion contact points [65]. Therefore, it is recommended for ceramic restorations, for monolith restorations in the case of the lateral parts of the arches, and for bistratified restorations in the frontal areas, where physiognomic aspects are most important. Thus, occlusal analysis with the T-Scan III system remains the gold standard for the assessment of dental occlusion, with multiple applications in dentistry.

A limitation of the study is represented by the number of participants included; however, four parameters were evaluated (maximum intercuspation, protrusion movement, and right and left lateral movements) with each system, which led to a set of 160 recordings to be compared. More clinical studies with the Medit I600 intraoral scanner are needed to improve the software and to ensure applicability in all important phases of dental treatment.

## 5. Conclusions

The objective of the present study was to establish the reliability of the Medit I600 scanner in the evaluation of dental occlusion, by comparing it with the T-Scan III scanner. The Medit I600 intraoral scanner, used for digital oral impressions, allows the transfer of the position of the two virtual models in occlusion (thus recording the occlusion); in terms of occlusal analysis, it provides data on the locations of the occlusal contacts, their surfaces, and the degree of interpenetration. The T-Scan III system provides data related to the distribution of the occlusal contacts, their surface areas, their amplitudes, and their dynamics over time. The locations provided by the two systems regarding the occlusal contacts at the tooth level correspond approximately, but the methods used for the evaluation of the occlusal contacts do not allow the precise assessment of their correspondence. For both systems, the highest values of occlusal contact are recorded for the position of maximum intercuspation, but with large differences in the surfaces of the occlusal contacts.

The T-Scan III system is considered the gold standard for occlusal analysis; therefore, more studies are needed to understand the data provided by the Medit I600 intraoral scanner, and medical practitioners should be aware of the limitations of the occlusal analysis provided by this system.

## Figures and Tables

**Table 1:** Pixel count after the evaluation of dental occlusion for maximum intercuspation, protrusion, right laterotrusion, and left laterotrusion.

No	Maximum Intercuspation						Protrusion						Right Laterotrusion						Left Laterotrusion					
	Red		Green		Blue		Red		Green		Blue		Red		Green		Blue		Red		Green		Blue	
	T-Scan	Medit	T-Scan	Medit	T-Scan	Medit	T-Scan	Medit	T-Scan	Medit	T-Scan	Medit	T-Scan	Medit	T-Scan	Medit	T-Scan	Medit	T-Scan	Medit	T-Scan	Medit	T-Scan	Medit
1	9	16	16	993	12,995	7403	0	0	0	9	812	636	0	140	0	3	365	448	0	0	0	25	340	260
2	50	36	110	36	10,638	1829	17	198	0	21	2285	861	8	73	0	10	1467	256	0	26	24	51	3567	680
3	33	265	52	1058	10,519	12,755	23	0	0	107	662	1088	24	0	136	108	389	1088	18	204	0	77	385	757
4	158	64	121	89	9856	2646	51	3	10	22	1413	1150	0	5	0	62	255	365	58	84	0	7	734	373
5	105	77	234	642	12,169	16,074	0	197	0	126	113	1621	0	1098	0	47	141	1628	0	21	0	13	213	385
6	35	0	650	30	13,034	1165	0	81	32	3	121	284	18	179	0	56	0	46	39	2352	0	36	336	661
7	12	69	62	36	9197	1618	0	50	130	11	777	759	25	0	0	21	1096	180	17	0	0	19	1708	366
8	62	12	120	436	7807	7383	0	0	32	145	540	1050	0	0	44	40	151	304	34	0	0	11	395	97
9	42	175	575	821	11,542	12,473	0	197	28	126	396	1621	0	1098	0	47	564	1628	24	21	62	13	1004	385
10	46	48	517	75	16,536	1786	0	25	0	4	171	83	0	5	0	36	163	27	0	0	0	0	109	20
11	214	26	112	236	17,975	3209	0	300	42	61	257	714	0	0	0	59	228	1141	0	185	60	7	301	114
12	44	321	407	1329	21,441	17,464	0	106	0	59	37	658	0	7	0	28	282	496	0	9	0	78	41	1370
13	9	16	16	993	12,995	7403	0	0	0	9	812	636	0	140	0	33	365	448	0	0	0	25	340	260
14	50	36	110	36	10,638	1829	17	198	0	21	2285	861	8	73	0	10	1467	256	0	26	24	51	3567	680
15	33	265	52	1058	10,519	12,755	23	0	0	107	662	1088	24	0	136	108	389	1088	18	204	0	77	385	757
16	158	64	121	89	9856	2646	51	3	10	22	1413	1150	0	5	0	22	255	365	58	84	0	7	734	373
17	44	321	407	1329	21,441	17,464	0	106	0	59	37	658	0	7	0	48	282	496	0	9	0	78	41	1370
18	42	175	575	821	11,542	12,473	0	197	28	126	396	1621	0	1098	0	47	564	1628	24	21	62	13	1004	385
19	46	48	517	75	16,536	1786	0	25	0	4	171	83	0	5	0	16	163	27	0	0	0	0	109	20
20	62	12	120	436	7807	7383	0	0	32	145	540	1050	0	0	44	40	151	304	34	0	0	11	395	97
Coeff/ *p* *	−0.132/ 0.432		−0.077/ 0.646		0.110/ 0.513		−0.094/ 0.622		0.235/ 0.195		0.144/ 0.394		−0.191 0.323		0.358/ 0.060		0.188/ 0.265		0.245/ 0.185		−0.025/ 0.897		0.099/ 0.555	

* Kendall’s tau-b test.

**Table 2:** Centralization of the values that numerically express the four variables—T-Scan III system.

Variable	Pixel	Minimum	Maximum	Mean ± SD	Median
MI position	Red	9	214	62.7 + 54.36	45
	Green	16	650	244.7 + 219.02	120.5
	Blue	7807	21,441	12,752.15 + 4023.67	11,542
Edge-to-edge protrusion position	Red	0	51	9.1 + 16.51	0
	Green Blue	0 37	130 2285	17.2 + 30.42 695 + 673.78	0 540
Edge-to-edge right laterotrusion position	Red	0	25	5.35 + 9.34	0
	Green	0	136	18 + 42.54	0
	Blue	0	1467	436.85 + 420.13	282
Edge-to-edge left laterotrusion position	Red	0	58	16.2 + 19.74	8.5
	Green Blue	0 41	62 3567	11.6 + 22.65 785.4 + 1032.02	0 385

**Table 3:** Centralization of the values that numerically express the four variables—Medit I600 intraoral scanner.

Variable	Pixel	Minimum	Maximum	Mean ± SD	Median
MI position	Red	0	321	102.3 + 109.07	56
	Green	30	1329	530.9 + 477.62	436
	Blue	1165	17,464	7477.2 + 5834.06	7383
Edge-to-edge protrusion position	Red	0	300	84.3 + 96.11	37.5
	Green Blue	3 83	145 1621	59.35 + 53.8 883.6 + 448.58	40.5 861
Edge-to-edge right laterotrusion position	Red	0	1098	196.65 + 392.39	6
	Green	1	108	30.8 + 32.42	22
	Blue	27	1628	610.95 + 546.14	406.5
Edge-to-edge left laterotrusion position	Red	0	2352	162.3 + 520.1	21
	Green Blue	0 20	78 1370	29.95 + 28.18 470.5 + 387.9	16 379

## Data Availability

The authors declare that the data of this research are available from the corresponding authors upon reasonable request.
